# Intestinal parasitic infections among children aged 7–14 years in Mizan-Aman city, Southwest Ethiopia: a community-based cross-sectional study

**DOI:** 10.3389/fpubh.2024.1478293

**Published:** 2024-12-24

**Authors:** Eyob Tekalign, Asresash Sebeta, Dejen Nureye, Tadesse Duguma, Tarkegn Tesfaye

**Affiliations:** ^1^Department of Medical Laboratory Sciences, College of Medicine and Health Sciences, Mizan-Tepi University, Mizan-Aman, Ethiopia; ^2^Southwest Ethiopian People Regional State Health Bureau, Dawro Zone, Ethiopia; ^3^School of Pharmacy, College of Medicine and Health Sciences, Mizan-Tepi University, Mizan-Aman, Ethiopia

**Keywords:** Intestinal parasitic infection, children aged 7-14 years, Mizan-Aman City, Southwest Ethiopia, community-based study

## Abstract

**Background:**

Intestinal parasitic infections continue to pose a major threat to human health globally, with a particularly high prevalence in developing countries. Soil-borne helminthiasis and schistosomiasis are notably widespread.

**Objective:**

The objective of the study was to determine the prevalence and contributing factors of intestinal parasites infection among participants aged 7–14 years.

**Methods:**

Community-wide prevalence study was undertaken from 30 August to 30 September 2021 in Mizan Aman Town. Socio-demographic information was collected using questionnaires. Three of the five kebels were randomly chosen. Households with children aged 7–14 were gathered from the chosen kebels and health post to recruit one eligible subject. Allocation of study subjects to each of the chosen kebels was computed proportionally. Two thick smear of Kato Katz technique was applied to examine stool samples. Data were entered and analyzed using SPSS version 20. To investigate the association between the dependent and independent variables, a logistic regression analysis was conducted. Statistics were considered significant for *p*-values under 0.05.

**Results:**

The overall prevalence of intestinal parasites was 64.6% (215/333). Of these, 51.05% (170/333) were infected with STHs, while 13.5% (45/333) had *S. mansoni*. *T. trichiura* was the most prevalent helminth. Infection intensity ranged from light to moderate was observed. Prior information about STHs (aORr:2.022 = CI:1.222–3.340), poor knowledge about STHs (aOR:1.677 = CI:1.057–2.660), unaware of deworming as prevention method of *S. mansoni* (aOR:2.620:CI:1267–5.418), swimming (aOR:0.448:CI:0.176–0.992) and contact with water (aOR:0.402:CI:0.169–0.957) were significantly associated with the *S. mansoni* infection.

**Conclusion and recommendation:**

The prevalence of intestinal parasite was high. Heavy infection was not recorded. Beyond mass deworming, the report emphasizes the necessity of ongoing public health interventions to address the high prevalence of these intestinal helminths.

## Introduction

Infection caused by intestinal parasites remains a significant global health issue, particularly affecting regions with high poverty rates, limited access to clean water, inadequate sanitation, and unsanitary living environments. In tropical regions where helminth infections are prevalent, it is not uncommon for individuals to acquire multiple types of gastrointestinal parasites, such as schistosomiasis and soil-transmitted helminthiasis, due to the high incidence of worm diseases (1, 2). The duration of infection, parasitic load, and type of species involved a role in determining the impact of intestinal worms. High levels of intestinal helminths and schistosomes in school-aged children can hinder their development, reduce their overall physical health, and impair their cognitive abilities (3).

The negative health effects mentioned above have a cumulative impact on the academic performance of children, leading to decreased school attendance (4). Additionally, it has been observed that hookworm infection (and potentially other diseases caused by parasitic worms) can hinder future earning potential (5). Moreover, hookworm and schistosomiasis are significant diseases that can affect pregnant women, resulting in premature births, lower birth weights, and increased health risks for mothers (6).

Globally, soil-transmitted helminths infection and schistosomiasis affect more than 1.5 billion individuals (24%) and 240 million individuals, respectively (7). Worldwide, there are over 267 million preschool-age children and 568 million school-age children residing in regions where STHs are highly prevalent and require treatment and preventive measures (8). In sub-Saharan Africa, approximately 198 million, 192 million, 173 million, and 162 million people are infected with hookworm, *Schistosoma* spp., *Ascaris lumbricoides*, and *Trichuris trichiura*, respectively (9, 10).

In Ethiopia, approximately 79 million individuals residing in areas where STHs are prevalent. This includes 9.1 million pre-school age children, 25.3 million school-age children, and 44.6 million adults above 18 years old. Moreover, an estimated 37.3 million people live in areas endemic to schistosomiasis, including 3.4 million pre-school children, 12.3 million school-aged children, and 21.6 million adults. According to estimates from 2013, the number of schistosomiasis cases in Ethiopia was approximately 35,775,100 (11). The overall pooled estimate of STHs in Ethiopia regions was reported to be 33%. The Southern Nations, Nationalities, and Peoples’ Region, Amhara Region, Oromia Region, and Tigray Region accounted for 44, 34, 31, and 10%, respectively (12). Ten years ago, a survey conducted in Mizan-Aman revealed a significant occurrence of intestinal parasites, with a prevalence rate of 76.7%. However, the research conducted at the institutional level failed to consider the environmental factors (13).

The government has been closely monitoring the prevention and control of these parasites, particularly in regions with high prevalence. In (14), the Federal Ministry of Health (FMOH) intensified its efforts to address schistosomiasis and STHs in regions where these diseases are prevalent, leading to the successful treatment of over 19 million people (15). Bench Sheko Health Department implemented school-based deworming activities by June 2023. Despite all efforts, there is often a lack of recent, localized data on the prevalence and distribution of these infections, making it difficult to design effective public health interventions. The purpose of this study was, therefore, to fill this gap by providing current data on the prevalence of intestinal parasite helminths, intensity and associated factors among this particular age group at the community level following deworming. This assessment can offer valuable insights for the development of a focused and effective strategy for prevention and control.

## Methods and materials

### Study setting, period and design

**Figure 1:**
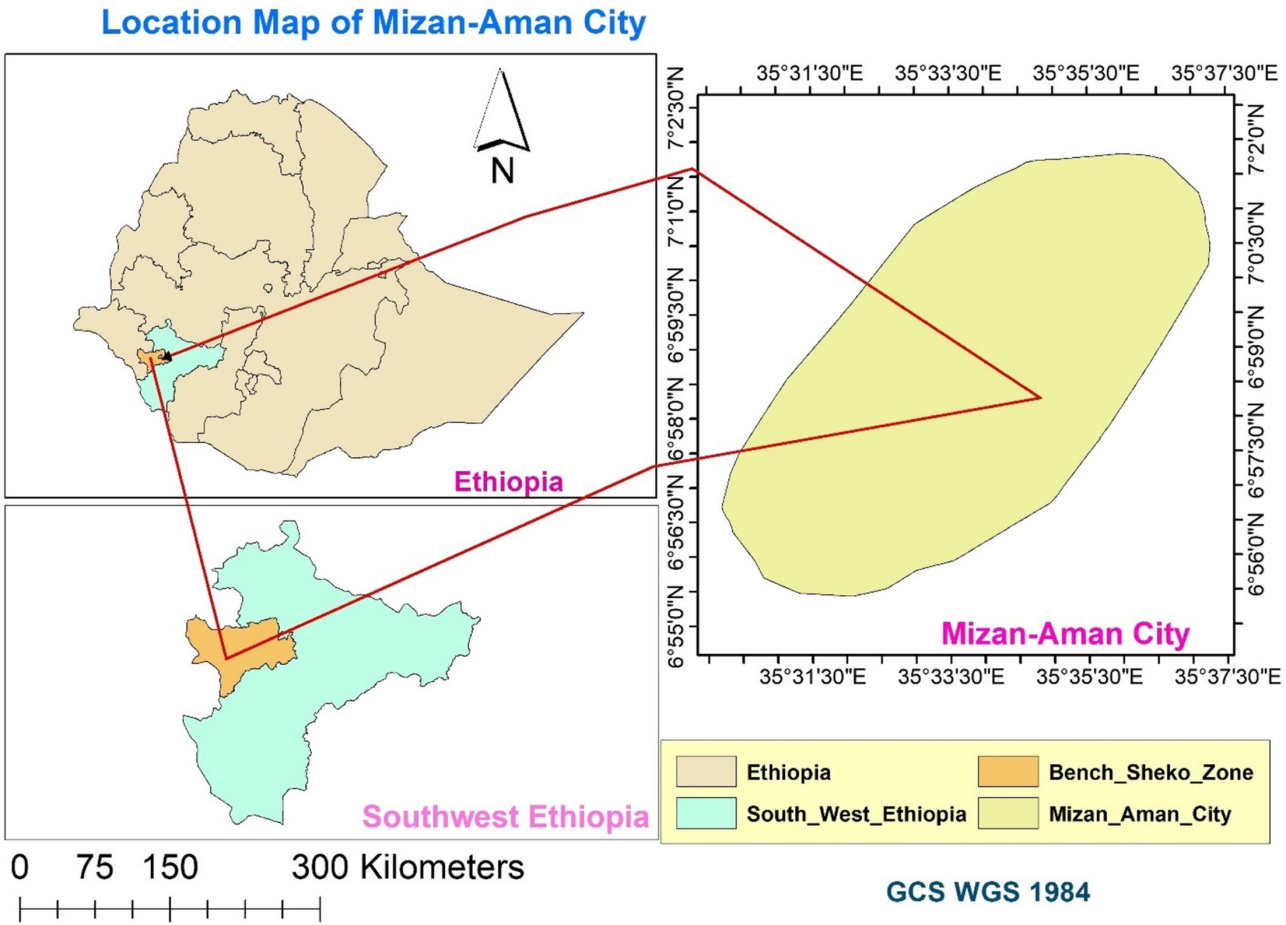
Map of the Study Area: Mizan-Aman City, Bench Sheko Zone, Southwest Ethiopian People’s Regional State.

A community-based cross-sectional study was carried out in Mizan Aman City from August 30 to September 30, 2021. Mizan-Aman city is situated in Bench Sheko Zone of the Southwest Ethiopian People’s Regional State. Twelve ethnic groups make up this region. The region consists of six Zones: Keffa, Sheka, Bench Sheko, Dawro, West Omo, and Konta (In the context of Ethiopia, a zone functions as a second-tier administrative division, situated below regions and above woredas, which are also known as districts). Bonga, Tercha, Mizan Aman, and Tepi are the four capital cities in the region. Although ethnic groups speak twelve different languages, Amharic is the working language in the region (16). Mizan-Aman city is located at an elevation of 1,451 meters above sea level and has latitudes between 6°55′0″N and 7°2′30″N and longitudes between 35°31′30″E and-35°37′30″E (Figure 1). The city is located 561 kilometers from Addis Ababa, the capital of Ethiopia, 50 kilometers from Tepi and 230 kilometers from Jimma. The city comprises five administrative kebels. The average yearly temperature in the area is 19.6°C. The annual rainfall is 2000 mm. The city is home to three higher education institutions—Mizan Tepi University, Aman Health Science College and Mizan ATVET College. In the city, clinical services are provided by the Mizan Aman Teaching Hospital, two health centers, and several private pharmacies and clinics (68).

### Population

The source population consisted of all individuals aged 7–14 residing in Mizan Aman city. The study population comprised children within the same age range, who were randomly selected from the designated Kebeles (the smallest and final level of administrative units in Ethiopia) based on specific inclusion criteria. These criteria required that the children had lived in these Kebles for at least 6 months and had not received any anthelmintic treatments (Albendazole, Mebendazole, Levamisole, Pyrantel pamoate and Praziquantel) for a duration of 2 weeks before the data collection commenced.

### Sample size determination

The sample size was calculated using methods suitable for a cross-sectional survey (18), based on a previously reported intestinal parasite prevalence of 73.9% (13). The calculation assumed a 15% non-response rate, a 5% margin of error, and a 95% confidence level. The initial sample size calculation resulted in 296 participants. After accounting for the 15% non-response rate (45 individuals), the final sample size was adjusted to 341 children.

### Sampling technique

Three kebeles (the smallest administrative units within a woreda or districts, which is the third level of administrative divisions next to Zone in Ethiopia) were selected at random from a pool of five kebels in the town. The households that were chosen had children between the ages of 7 and 14, and this selection was based on the registration records and family folders that were accessible at the health posts. In these three Kebels, there was a combined total of 2053 children. Specifically, there were 691 children in Hibert Kebel, 609 children in Kometa Kebel, and 753 children in Addis Ketema Kebel. The selection process involved allocating and sampling children in a proportional manner. Specifically, 112 children from Hibert Kebel, 99 children from Kometa Kebel, and 122 children from Addis Ketema Kebel were chosen. In cases where a household had only one child between the ages of 7 and 14, that child was selected. However, if there were multiple children within this age range, one child was randomly chosen through a lottery method.

### Questionnaire survey

The questionnaire was written primarily in English and translated into Amharic for ease of understanding. A comparison was made regarding the consistency of the two versions. Three skilled data collectors conducted face-to-face interviews to gather the data. A preliminary study was conducted using 5% of the study subjects who were not from the study area but possessed comparable attributes in order to facilitate the required adjustments. All ambiguous inquiries and duplicated concepts were rectified. Sociodemographic factors and risk elements linked to STH and *S. mansoni* were gathered through a questionnaire. A survey was conducted to evaluate the type and condition of latrines, the existence of waste disposal sites, the occurrence of open defecation near the residence, the presence of animals, and the hygiene of the child’s fingernails.

### Parasitological examination

Approximately 5 grams of fresh fecal specimens were gathered utilizing labeled containers. The specimens were then stored in an ice box and transported to Mizan-Tepi University Parasitology Laboratory for processing and examination. The protocol of Kato-Katz was as follows: Fresh fecal sample was filtered through a mesh sieve to remove larger particles. A total of 41.7 mg of the strained stool was transferred on a microscope slide using 41.7 mg template. One pieces of cellophane soaked in a glycerol-malachite green solution for 24 h was placed on a slide. In order to produce a smear, the cellophane was pressed down with another microscope slide. Two thick smears were prepared from a single stool sample. The slides were then examined under a microscope within a time frame of 40–60 min after preparation to prevent the loss of hookworm parasites. Eggs in each prepared slide were counted and multiplied by a factor 24 to express the eggs per gram (EPG) of feces (19). Species-specific infection intensity categories had been classified as mild, moderate, and severe according to thresholds set by the World Health Organization (20).

### Quality assurance

The data collectors and the laboratory technologist underwent training in collecting questionnaire data and utilizing Kato-Katz techniques. The data collectors were supervised during the data collection process. The Principal Investigator confirmed the questionnaire’s completeness and the overall activities in the laboratory. Reagent and smear preparation for microscopic examination were reviewed. The egg quantification of 10% of post-analyzed slides was re-evaluated by an experienced laboratory technician and the Principal Investigator.

### Date processing and analysis

The data analysis underwent a process of data validation, data cleaning, editing, coding, inputting, and analyzing using SPSS version 20. The prevalence of soil-transmitted helminthiasis and schistosomiasis infection was reported in percentage. Logistic regression analysis was conducted to examine the potential significant associations. The potential candidates for multivariate analysis were determined by selecting the independent variables with a *p*-value ≤0.2 in bivariate analysis. In the multivariate analysis, the adjusted odds ratios (aOR) with a 95% confidence interval (CI) and a *p*-value <0.05 were considered to be statistically significant.

### Operational definition

In our research context, an operational definition refers to how a variable defined for the purposes of the study.

**Prior Information (heard) about Helminths**: we are specifically referring to whether an individual had been previously informed or not about helminth infections before evaluating their knowledge based on the questions, we had set based on the criteria listed below:

**Previously informed (Prior information)**

If participants who can correctly answer at least one or more questions about STHs, such as:

- Can they identify at least one type of helminth?
- Do they know at least one method of preventing STHs?
- Have they heard of deworming treatments and their importance?
- Have they ever been exposed to information about helminths through health education programs, schools, media, or community health outreach?

**Not previously informed (no prior information)**

If participants who were unaware of key aspects of helminths and their prevention, such as:

- Unable to identify any specific helminth species or transmission routes.
- Not familiar with basic prevention methods like handwashing or the use of latrines.
- Not previously exposed to educational messages about helminths.

**Good knowledge**: refers to those who answered and scored greater than the mean value of all the question we had set to assess the participants knowledge.

**Poor knowledge**: refers to those who answered and scored less than the mean value of all the question we had set to assess the participants knowledge.

**Woredas (Districts)**: are the third level of administrative divisions in Ethiopia, following zones and regional states.

**Keble**: The smallest and final level of administrative units in Ethiopia.

**Animals**: refers to animals play vital roles in Ethiopian households, particularly in rural areas, where they contribute to the family’s livelihood, transportation, and food security.

## Results

### Socio-demographic characteristics of the study subjects

The household survey revealed that 333 school children participated, with a response rate of 97.6%. Of these, 187 (56.2%) were male, and the majority, 186 (55.9%) fell within the age range of 7–10 years old. The average age was 10.4 ± 1.2 years old. Furthermore, 184 (55.3%) children were in school grades 1–4. The educational status indicated that 253 fathers (76%) and 211 mothers (63.4%) were able to read and write (Table 1).

**Table 1:** Socio-demographic characteristics of study participants in Mizan-Aman City, Bench Sheko Zone, Southwest Ethiopia, 2021.

Variables	Categories	Frequency	Percent
Age group	7–10	186	55.9
	11–14	147	44.1
Sex	Male	187	56.2
	Female	146	43.8
Grade level	1–4	184	55.3
	5–8	149	44.7
Father education	Illiterate	80	24
	Read and write	253	76
Mother education	Illiterate	122	36.6
	Read and write	211	63.4
Father occupation	Farmer	173	52
	Merchant	66	19.8
	Employee	56	16.8
	Daily laborer	38	11.4
Mother occupation	Housewife	245	73.6
	Merchant	55	16.5
	Employee	26	7.8
	Daily laborer	7	2
Family size	<5	147	44.1
	≥5	186	55.8
Residence	Urban	188	56.4
	Rural	145	43.5
School type	Government	244	73.3
	Private	89	26.7
Animals at home	Yes	188	56.4
	No	145	43.6

### Soil transmitted helminths and *Schistosoma* infection related to sanitation

Among the study participants, 308 individuals (92.5%) possessed a toilet in their homes, while 249 individuals (74.8%) consistently utilized their home toilets. It was observed that 107 children (32.4%) had a habit of defecating in open fields. Additionally, 233 children (69.9%) engaged in swimming activities. As for hygiene practices, 247 children (74.2%) occasionally used soap and water after using the toilet (Table 2).

**Table 2:** The distribution of soil transmitted helminths and S. mansoni infection in related to respondent sanitation practices, Mizan-Aman City. Bench Sheko Zone, Southwest Ethiopia, 2021.

Variables	Categories	No, %	STH, %	*S. mansoni*, %
Latrine at home	Present	308 (92.5)	158 (47.4)	42 (12.6)
	Absent	25 (7.5)	12 (3.6)	3 (0.9)
Latrine utilization	Always	239 (71.8)	125 (37.5)	36 (10.8)
	Sometimes	94 (28.2)	45 (13.5)	9 (2.7)
Open defecation	Yes	108 (32.4)	56 (18.8)	14 (4.2)
	No	225 (67.6)	114 (34.2)	31 (9.3)
Fruit washing	Yes	145 (43.5)	60 (18.0)	22 (6.6)
	No	188 (56.4)	110 (33.0)	23 (6.9)
Washing with Soap & water after using toilet	Yes	247 (74.2)	155 (34.4)	34 (10.2)
	No	86 (25.8)	55 (16,5)	11 (3.3)
Washing hands before meal	Always	219 (65.8)	103 (30.9)	30 (9.0)
	Sometime	114 (34.2)	67 (20.1)	15 (4.5)
Habit of swimming	No	100 (30.0)	63 (18.9)	16 (4.8)
	Yes	233 (70.0)	107 (32.1)	29 (8.7)
Habit of wearing shoes	Always	93 (27.9)	40 (12.0)	9 (2.7)
	Sometimes	240 (72.1)	130 (39.0)	36 (10.8)
Cut fingernails	No	127 (38.1)	108 (19.2)	18 (2.4)
	Yes	206 (61.9)	62 (31.8)	27 (8.1)
Nail biting habit	No	215 (64.6)	97 (29.1)	28 (8.4)
	Yes	118 (35.4)	72(21.6)	17(5.1)
Drinking water source	Pipe	165 (49.5)	79 (23.7)	26 (7.8)
	Well	26 (7.8)	18 (5.4)	4 (1.2)
	Stream	142 (42.6)	73 (21.9)	15 (4.5)
Waste disposal	Present	178 (53.5)	95 (28.5)	21 (6.3)
	Absent	155 (46.5)	75 (22.5)	24 (7.2)

### Prevalence of soil transmitted helminths and *Schistosoma mansoni*

The combined prevalence of soil-transmitted helminths (STHs) and *S. mansoni* was 215 (64.6%). Specifically, 170 individuals (51.05%) were infected with STHs, while 45 individuals (13.5%) were infected with *S. mansoni.* A total of five different types including *S. mansoni* were identified during the study. *T. trichiura* was the most common parasite, infecting 97 individuals (29.1%), followed by *Ascaris lumbricoides* with 64 case (19.2%), and *S. mansoni* with 45 cases (13.5%). The age group most affected with both groups by the consisted mainly of children aged 7–10 years old. Among the 333 students who took part in the study, 165 (49.5%) had a single infection, while 50 (15.0%) were co-infected with more than one helminth (Table 3).

**Table 3:** Infection status of respondents by age distribution in Mizan-Aman City, Bench Sheko Zone, Southwest Ethiopia, 2021.

Infection status	Age group					
	7 to 10	%	11 to 14	%	Total	(%)
*Trichuris trichiura*	61	18.3	36	10	97	29.1
*Ascaris lumbricoides*	36	10	28	8.4	64	19.2
Schistosoma mansoni	18	5.4	27	8.1	45	13.5
Ancylostomatide gen. sp.	3	0.9	5	1.5	8	2.4
*Hymenolepis nana*	1	0.3	0	0	1	0.3
Co-infection	33	9.9	17	5.1	50	15
Single infection	131	39.3	34	10.2	165	49.5
No helminths	56	16.8	62	18.6	118	35.4

### Co-infection with intestinal helminths

This study also found that multiple intestinal helminths can coexist in the same individuals. Among the 50 study participants, the most common co-infection was *A. lumbricoides* and *T. trichiura*, which occurred in 56% (28/50) of respondents. Other significant co-infections included *A. lumbricoides* and *S. mansoni* (16%), *T. trichiura* and *S. mansoni* (14%), *A. lumbricoides*, *T. trichiura* and *S. mansoni* co-infection (8%), and Hookworm and *T. trichiura* (6%).

### Helminth infection intensity and distribution

The study found that the majority of participants who tested positive for STH and *S. mansoni* had low-level infections. Specifically, the Kato-Katz detection method 253 showed that out of 170 individuals, 85.3% (145) had mild infections and 14.1% (24/170) 254 had moderate infections of soil-transmitted helminths. As for *S. mansoni*, 82.2% 255 (37/45) had light infections and 17.8% (8/45) had moderate infections (Table 4).

**Table 4:** Intensity of STHs and *S. mansoni* infection among study participants in Mizan-Aman City, Bench Sheko Zone, Southwest Ethiopia, 2021.

Types of helminths	EPG (GM)	Light infection	Moderate infection
*T. trichiura*	983/gram	83 (24.9%)	14 (4.2%)
*A. lumbricoides*	6,078/gram	57 (17.1%)	7 (2.1%)
Hookworm	606/gram	5 (1.5%)	3 (0.9%)
	Overall STH intensity	**145 (85.3%)**	**24 (14.1%)**
*S. mansoni*	301/gram	**37 (82.2%)**	**8 (17.8%)**

The bold and * indicate the variable significantly associated with STHs infection at *p*-value <0.05.

### Factors associated with soil transmitted helminths

Using the information from the table, the parameters linked to the existence of soil-transmitted helminth (STH) infections were analyzed. Crude odds ratios (COR), adjusted odds ratios (aOR), and *p*-values for the relationships between different independent variables and infection status were all included in the analysis. To choose the candidate, the COR was examined. The parameters linked to the STHs infection were revealed by the adjusted odd ratio with *p* < 0.05. Notably, children who had not never heard of STHs before (*p* = 0.006) and children with poor knowledge about STHs (*p* = 0.028) were significantly associated with increased infection (Table 5).

**Table 5:** Bivariate and multivariate analysis of STHs infection among children of Mizan- Aman City, Bench Sheko Zone, Southwest Ethiopia, 2021.

Variables	Infection status		COR (95% CI)	aOR (95%CI)	*p*-value
	Positive, %	Negative, %			
Gender					
Male	89 (26.7)	98 (29.4)	Ref	Ref	0.183
Female	81 (24.3)	65 (19.5)	1.372 (0.889–2.119)	1.369 (0.862–2.172)	
Age grouped					
7–10	101 (30.3)	85 (25.5)	Ref	Ref	0.381
11–14	69 (20.7)	78 (23.4)	0.744 (0.482–1.149)	0.813 (0.512–1.292)	
Mother education					
Not read &write	68 (20.4)	54 (16.2)	Ref		0.923
Read and write	102 (30.6)	109 (32.7)	0.743 (0.475–1.163)	1.026 (0.606–1.737)	
Father education					
Not read &write	49 (14.7)	31 (9.3)	Ref	Ref	0.544
Read and write	121 (36.3)	132 (39.6)	0.580 (0.347–0.960)	0.833 (0.462–1.503)	
Residence					
Urban	102 (30.6)	86 (25.8)	Ref	Ref	0.225
Rural	68 (20.4)	77 (23.1)	0.745 (0.482–1.150)	0.748 (0.468–1.196)	
Washing fruits					
No	60 (18)	78 (23.4)	Ref	Ref	0.079
Yes	110 (33)	85 (25.5)	0.501 (0.322–0.777)	0.618-(0.3611.057)	
Soap & water after toilet use					
No	55 (16.5)	31 (9.3)	Ref	Ref	0.167
Yes	115 (34.5)	132 (39.6)	0.491 (0.296–0.815)	0.651 (0.354–1.196)	
Hands washing before meal					
Always	103 (30.9)	116 (34.8)	Ref	Ref	0.432
Sometimes	67 (20.1)	47 (14.1)	1.605 (0.016–2.537)	1.241 (0.725–2.124)	
Shoes wearing					
Always	40 (12.0)	53 (15.9)	Ref	Ref	0.812
Sometimes	130 (39.0)	110 (33.3)	1.566 (0.966–2.537)	1.071 (0.608–1.887)	
Open defecation					
Sometimes	56 (16.8)	52 (15.6)	Ref	Ref	0.290
Not at all	114 (34.2)	111 (33.3)	1.505 (0.946–2.395)	1.309 (0.795–2.155)	
Previous heard about STH					
No	127 (12.9)	104 (17.7)	Ref	Ref	0.006*
Yes	43 (38.1)	59 (31.2)	1.676 (1.046–2.683)	2.022 (1.222–3.346)	
Knowledge on STHs					
Poor	108 (32.4)	83 (24.9)	Ref	Ref	0.028*
Good	62 (18.6)	80 (24.0)	1.679 (1.084–2.602)	1.677 (1.057–2.660)	

The bold and * indicate the variable significantly associated with STHs infection at *p*-value <0.05.

### Factors associated with *Schistosoma mansoni*

It was evident in our research that participants who were unaware of deworming as a preventative and control presented an odds of being infected 2.6 times higher than children who understood that deworming was a means of control and prevention. Children who engaged in swimming had a 99.5% higher chance of contracting *S. mansoni*. Children who knew that *S. mansoni* is transmitted through contact with fecally contaminated water were 99.6% less likely to be infected with *S. mansoni* (Table 6).

**Table 6:** Bivariate and multivariate analysis and associated factors of S. mansoni infection among children of Mizan-Aman City, Bench Sheko Zone, Southwest Ethiopia, 2021.

Variables	Infection status		COR (95% CI)	aOR (95%CI)	*p*-value
	Positive, (n, %)	Negative, (n, %)			
Open defecation					
No	18 (5.4)	82 (24.6)	Ref	Ref	0.883
Yes	27 (8.1)	206 (61.9)	1.695 (0.886–3.246)	1.156 (0.507–2.20)	
Prevention by deworming					
No	29 (8.7)	110 (33.0)	Ref	Ref	0.009*
Yes	16 (4.8)	178 (53.4)	2.933 (1.523–5.647)	2.620 (1.267–5.418)	
Swimming practice					
No	7 (2.1)	93 (27.9)	Ref	Ref	0.048*
Yes	38 (11.4)	195 (58.5)	1.346 (0.860–2.106)	0.448 (0.176–0.992)	
Transmission via water contact					
No	36 (10.8)	261 (78.4)	Ref	Ref	0.039*
Yes	9 (2.7)	27 (8.1)	0.580 (0.347–0.960)	0.402 (0.169–0.957)	

The bold and * indicate the variable significantly associated with *S. mansoni* infection at *p*-value < 0.05.

## Discussion

There is still a public health concern regarding soil-transmitted helminthiasis and schistosomiases, although there has been a significant difference in their distribution. The transmission of the infection occurs through water and soil contaminated with human waste. This research aimed to showcase the prevalence and intensity of infection of STHs and *S. mansoni*, individually. The combined prevalence of STHs and *S. mansoni* was 64.9%. Meanwhile, STHs make up a total prevalence of 170 (51.1%). It is believed that the local factors identified in the study area contributed to creating favorable conditions for this prevalence. Our study’s results also showed that *T. trichiura* was the most common helminth, with light to moderate infection intensity. *A. lumbricoides* and hookworms were next, with comparable infection intensity levels. The findings of a previous study conducted in the same area also emphasized this concern, reporting infection intensity ranging from light to heavy. However, our study found a lower prevalence, with infection intensity primarily categorized as light to moderate (13). The reduction in overall prevalence and the difference in intensity range might be attributed to the government’s focus on implementing the neglected tropical disease prevention and control program (69). However, the continued prevalence reflects the issue and underscores the need for public health intervention in these resource-limited settings. The documented results indicated that, based on the World Health Organization’s risk classification, the study subjects were categorized as high-risk, with an infection rate of 50% or higher (21).

In this study, the prevalence of *T. trichiura* and *A. lumbricoides* decreased with age; however, age was not identified as a significant associated factor, which is consistent with the findings of a previous study (22). Similarly, another study (23) also observed that age was associated with a reduction in both the likelihood of infection and the severity of any soil-transmitted helminth (STH) infection. However, as people aged, the prevalence of *S. mansoni* and Hookworm infections rose. Spending more time in high-risk environment, participation in high-risk activities like playing barefoot or swimming in tainted water might be contributing factors. The relationship between age and the risk of acquiring these infections is complex and can vary based on factors like immune response, behavior, environmental exposure, and access to public health interventions. Intestinal helminths infections are typically associated with poor sanitation, contaminated water, and soil, but their patterns of transmission and the age groups most at risk can differ.

The prevalence in our study is lower than that reported in Indian studies, where it stands at 75.6%, with *A. lumbricoides* being the most STHs and the majority of infections being of low intensity (24). The higher prevalence in India might be attributed to several factors, including the large sample size, risk variables similar to those in our study, and the absence of a deworming program in India prior to (14) during the time of the investigation. In Runda, the prevalence of STH infections (77%) is higher than in our study, with *T. trichiura* being the most commonly observed species. The study also found moderate to high-intensity infections of *T. trichiura* and *A. lumbricoides* (25). Various factors, including altitude, soil types, temperature, the level of awareness among participants, differences in sample sizes, the season during which the study was conducted, study designs might be contributed to these observed variations.

The prevalences in Bahir Dar City, Mettu Town, Yachi, and Zarima were higher than in our study, with rates of 85.9% (26), 84.4% (27), 70.7% (28), and 82.4% (29), respectively. In those areas, hookworm, *A. lumbricoides*, *S. mansoni*, and *A. lumbricoides* were the most commonly identified species, in that order. The differences in prevalence might be attributed to factors such as variations in sample sizes, lack of awareness and the presence water bodies used by residents for irrigation and household activities. However, comparable results were observed in Gonder, 66.7% (30), Hawassa Tula Sub-City, 67.7% (31), Adwa (Tigray), 58.8% (32), and West Hararghe, 61.5% (33). The figures implicated that intensity of soil-transmitted helminths and prevalence are clearly influenced by ecological conditions, which could explain the differences observed (34).

Although *T. trichiura* and *A. lumbricoides* were the most prevalent species in Indonesia, the Indonesian data showed that the prevalence of STHs was lower and that the severity of STH infection ranged from mild to severe. The results variation might be linked to personal, environmental aspects (35), exposure and small number of study participants involved. In Afghanistan, a polyparasitic infection involving soil-transmitted helminths (STHs) and a low prevalence of intestinal parasites (39.8%) was reported. In contrast to our findings, *A. lumbricoides* was the most common causative agent. One potential contributing factor identified was living in mud homes, which increased the risk of poor clearance of parasite eggs and facilitated the spread of infection. Additionally, the study’s large sample size likely contributed to the higher number of infected children compared to our study, even though the overall prevalence was low (17). Studies from other countries had reported varying lower prevalences of soil-transmitted helminthiasis, 9.3% in China (36), 37% in Malaysia (37), 30% in Nigeria (14), 12.95% in Kenya (38), and 32% in Tajikistan (39), all with low infection intensity. These lower prevalences and intensities may be linked to sociodemographic factors as well as the implementation of deworming programs as a control measure.

Other studies had shown that STH infections can lead to significant health issues. In Gonder, 32.3% of patients were reported to have STHs, with *Ascaris lumbricoides* being the predominant species, ranging in intensity from mild to high. In contrast, other helminths were associated with mild to moderate infections (40). In The prevalence in Hawassa City, Ethiopia, was 49.7% (67), and the lower rate in schools may be attributed to intervention programs. Most study participants in Jimma exhibited light infection intensity, with a prevalence of 45.6% (42). The differences in detection sensitivity between the McMaster and Kato-Katz methods, which are based on fecal egg counts, might explain the study’s lower magnitude and infection intensity. Numerous studies conducted in our country had reported a variety of dominant species, with prevalences ranging from 9.5 to 36.2%, typically with light infections, though some cases show very low to moderate heavy intensity infections (40, 43–47).

In the tropics and subtropics, especially in sub-Saharan African nations like Ethiopia, schistosomiasis is a prevalent helminthic infection. *S. mansoni* infection is a serious public health issue in these counties despite multiple rounds of preventative treatment, there is a chance of reinfection and recurrent illness. Age, water body contact habits, and gender all affect the frequency of *S. mansoni* in Ethiopia. By 2030, the nation aimed to stop the spread of several neglected tropical diseases, such as schistosomiasis. To meet the goals set by the nation, an epidemiological survey with updated findings is therefore necessary. In this investigation, the prevalence of *S. mansoni* was 13.5%, and the infection severity ranged from mild to moderate. The finding suggested that schistosomiasis remains a significant public health concern.

Studies conducted in various regions of Ethiopia had reported prevalence rates ranging from 20 to 89.9%, with infection intensity varying from low to heavy (29, 41, 48–53). In other countries such as Tanzania, Kenya, and Madagascar, prevalence ranged from 41.3 to 73.2%, with infections ranging from light to heavy, although no cases of heavy infection were reported in Tanzania (38, 54, 55). These variations in prevalence and intensity may be attributed to several factors, including altitude, temperature (which favors the multiplication and survival of the intermediate host), water contact behaviors, snail density, proximity of schools and homes to rivers (which may increase contact), sample size, occupation (e.g., fishing and irrigated farming), and the level of local awareness.

Our finding is comparable to those reported from other endemic areas, such as Guangua District (12.6%) (56), Wondo District (11.4%) (53), the Niger River basin (12.2%) (57), and Dembia (15.4%) (58). This similarity may be attributed to common recreational practices and the endemic nature of these areas. In contrast, lower prevalences were observed in Sudan (5.9%) (59) and Bamako, Mali (1.5%) (60). These could be due to less contamination of water bodies with feces, a lower distribution of the intermediate host, and the use of single egg counts in parasite examination. Our study, which used two slides per child, likely provided a more accurate estimate of both the prevalence and intensity of *S. mansoni*.

The participants in this study were found to have limited knowledge about the prevention, transmission, and control of soil-transmitted helminths (STHs). This lack of knowledge was associated with a higher likelihood of STH infection. It is consistent with a previous study conducted in the Lake Tana region, which also observed higher infection rates among individuals with limited awareness of prevention and control measures (26). Other study had also shown that children with inadequate knowledge are at a reduced risk of soil-transmitted helminth (STH) infections (61). In Nigeria, children who did not have prior knowledge about soil transmitted helminths infections had high likelihood of infection (62). Our findings align with this observation. This phenomenon may be attributed to a lack of awareness among parents about how to prevent, transmit, and control STH infections. As a result, this knowledge gap leaves children poorly informed, leading to higher infection rates, as also observed in study from Bangladesh (63). The analysis of multiple variables showed that engaging in swimming, having insufficient knowledge about preventing and controlling *S. mansoni* through deworming, and the transmission of the disease through contact with water contaminated with feces were all linked to *S. mansoni* infection. Studies conducted in Mekelle City (64), Tanzania (55), Wolaita (53), Zarima (29), Gorgora (65), Jimma (42) and Sanja, students’ behaviors related to water activities were identified as risk factors for *S. mansoni* (50). However, in contrast, study in the Gambia indicated that swimming and bathing in bodies of water presented a lower risk, suggesting that children’s actual water interaction behavior may not be fully captured (66).

### Strength

The strength of a cross-sectional study lies in its ability to rapidly assess the prevalence and distribution of these infections, identify risk factors, and provide essential baseline data for public health planning. It is a powerful tool for guiding both immediate interventions and long-term strategies to combat these neglected tropical diseases in vulnerable populations. There are various benefits to using the double Kato-Katz procedure, which entails extracting two distinct stool smears from the same sample and running the test twice. The accuracy and dependability of the study’s findings are increased by these benefits, which include increased sensitivity, accuracy, and overall efficacy in identifying and measuring helminth infections, especially in regions with low-intensity infections, multiple species, and when eggs are unevenly distributed in the stool sample (Reduce false negative). This is especially crucial when assessing the efficacy of public health initiatives, such deworming campaigns, when accurate prevalence and severity data are essential. Additionally, the study underscored its dedication to participant welfare by promptly referring individuals diagnosed with intestinal parasitic infections to local health facilities for suitable treatment.

### Limitation

Cross-sectional studies have many advantages, but it’s crucial to remember that they are correlational rather than causative. Since the data is gathered all at once, the study is unable to monitor the beginning or progression of infections over time, making it impossible to determine cause-and-effect links.

## Conclusion and recommendation

The results of this community-based cross-sectional study revealed a high prevalence of STHs (≥50 WHO threshold) and a moderate risk of morbidity caused by *S. mansoni*, with a prevalence greater than 10% but less than 50%, according to the WHO threshold. Both infections were found to be associated with factors such as Lack of prior information about STHs and poor knowledge with regard to the transmission, prevention, and control methods. Regarding *S. mansoni*, the identified associated factors included lack of knowledge about its transmission through contact with fecally contaminated water and unawareness of deworming as a preventive measure and swimming habit. The study emphasizes the importance of ongoing public health interventions to address the issue. Despite current deworming programs, the continued presence of these infections highlights the need for comprehensive strategies that not only focus on treatment but also address the environmental and behavioral factors contributing to transmission.

## Data Availability

The original contributions presented in the study are included in the article/supplementary material, further inquiries can be directed to the corresponding author.
